# Supplementary material for: Genomic and functional analysis of Romboutsia ilealis CRIBT reveals adaptation to the small intestine
Source: PeerJ. 2017 Sep 11;5:e3698. doi: 10.7717/peerj.3698 (PMC5598433; doi:10.7717/peerj.3698)
Supplement: Supplemental Information 1 — Additional information from the materials and methods, which were shortened in the main manuscript. [file peerj-05-3698-s001.docx]

**Text S1 for**

# **Genomic and functional analysis of *Romboutsia ilealis* CRIB^T­^_­_ reveals adaptation to the small intestine**

Jacoline Gerritsen^*^_,_ Bastian Hornung^*^, Bernadet Renckens, Sacha A. F. T. van Hijum, Vitor A.P. Martins dos Santos_,_ Ger T. Rijkers, Peter J. Schaap, Willem M. de Vos and Hauke Smidt

**Supplemental Methods**

**Genomic DNA extraction**

Genomic DNA was extracted from overnight grown cultures of *Romboutsia ilealis* CRIB^t^. Cells were pelleted by centrifugation at 9400 × g for 10 min and directly used for DNA extraction. DNA quality and concentrations were determined using a NanoDrop ND-1000 spectrophotometer (NanoDrop Technologies, Inc, Wilmington, DE, USA) spectrophotometric analysis and by electrophoresis in a 1.0 % (w/v) agarose gel. DNA was stored at 4 °C until subsequent sequencing.

**Genome sequencing, assembly and annotation**

The total sequence data amounted to 234,223 reads from the pyrosequencing, 34,807,392 Illumina paired-end reads (50 bp long), 9,886,736 Illumina mate-pair reads (4kb insert, 50 bp long) and 3699 PacBio circular consensus sequencing (CCS) reads (1).

For genome assembly, the size of the genome was predicted with KMERSPECTRUMANALYZER (downloaded at 15.08.2013) (2). The assembly was done in parallel using two different assemblers. Ray v2.2 (3) was used for the untrimmed Illumina paired-end dataset together with the Illumina mate-pair and PacBio CCS datasets. Default settings were used with the exception of increasing the k-mer length to 35 bp. In addition, Edena v3.130110 (4) was used on the Illumina paired-end and mate-pair datasets using default settings. The contigs obtained with both assemblers were merged using Zorro, the masked assembler (http://lge.ibi.unicamp.br/zorro/; release 13.04.2011), which relies on alignments with Nucmer (5) to determine duplications and misassembled contigs. The merge was done with default settings, with input of one of the paired-end files of the Illumina paired-end dataset. Scaffolding was done with the merged contigs using Opera v1.2 (6) using default settings besides the usage of bowtie v1.0.0 (7), and the paired- end data as input, and afterwards further scaffolding was done with the mate-pair dataset using SSPACE v2.0 (8). Default settings were used together with an expected insert size of 4000 nucleotides and an error of 0.5. After the scaffolding step with opera, all contigs were discarded that either had a length of less than 100 bp or which mapped to another region of a bigger contig with 99 % identity over 98 % of its length. To close the gaps in the resulting assembly, GapFiller v1.11 (9) was used with the Illumina paired-end and mate-pair reads. Insert size for the Illumina paired-end data was estimated by mapping the raw Illumina paired-end reads back to both the initial assemblies using Bowtie2 v2.0.6 (10) and the CollectInsertSizeMetrics utility from the PicardTools package v1.94 (http://picard.sourceforge.net/). Default settings were used with exception of increasing the number of iterations to 40. Next, two further rounds of scaffolding using SSPACE and gapfilling using GapFiller were performed as described above. After the second round of gapfilling, all scaffolds with a length of less than 500 bp were discarded. As last step in the assembly, Pilon v1.4 (http://www.broadinstitute.org/software/pilon/) was used for quality assurance using Illumina paired-end and PacBio CSS reads mapped to the assembled genome with Bowtie2 v2.0.6 (10) using default settings. Afterwards a final round of gapfilling was performed.

To control the quality of intermediate and final assemblies, intermediate steps were annotated, and all predicted proteins were compared to the predicted proteome of *R. ilealis* CRIB^T^ itself by BLASTP analysis. If no unlikely duplications (multiple co-located proteins with more than 95 % identity, which are unlikely to be of biological origin, in contrast to e.g. transposases) as a result of one of the last steps in the assembly were identified, the assembly was used for further refinement. To exclude that any duplication was missed during the annotation, also a TBLASTX search of protein coding sequences against the genome was performed. Additional quality checks were done on duplications of single copy genes (11), completeness of rRNA operons and the presence of all tRNAs. To control the intermediate steps for possible loss or duplication of genomic material, the Mauve aligner (12) was used to compare the assemblies resulting from the different steps described above against each other, as well as the BLAST Ring Image Generator (13).

CDS were assigned to clusters of orthologous groups (COGs) via bidirectional best hit (14) against the COG database (15) applying an e-value cut-off of 0.0001. A further step of automatic curation was performed, by weighting the annotation of the different associated domains, and penalizing uninformative functions (e.g. ‘Domain of unknown function’), and prioritizing functions of interest (e.g. domains containing ‘virus’, ‘phage’, ‘integrase’ for phage related elements; similar procedure for different other functions). Pseudogenes were identified by manual curation of genes of interest. Signal peptides were predicted with SignalP v4.1 (16).

### Metabolic modelling

Pathway tools v18.0 (17) was used on the annotation to build a genome-scale metabolic model, which was manually curated with the built in curation tools. Afterwards a flux balance analysis (FBA) was performed with the integrated FBA tool. The necessary biomass components were obtained from related literature of genome-scale metabolic models for *Mycoplasma genitalium* (18) and *Staphylococcus aureus* (19). Pathways for the production of essential cofactors were manually checked using the CoFactor database (20). All carbohydrates previously tested *in vitro* were tested in the FBA, to verify whether they could serve as energy and carbon source.

The initial medium consisted out of the mentioned carbohydrate sources, urea/NH_3_, SO_3_, Pi and H_2_S, and was subsequently during testing expanded with all the biomass components, for which auxotrophies exist in the genome (e.g. amino acids and vitamins/cofactors).

The pathway tools database, including the .fba file for the FBA simulations, has been included as supplementary file 1.

**Carbohydrate growth experiment**

For the carbohydrate growth experiment cultures were three times transferred on the respective carbohydrate before start of the experiment. As control, basal medium (without an additional carbohydrate) was inoculated with cells preconditioned on each of the carbohydrates (one culture for each carbohydrate). Cell pellets of 2 mL cultures were used for RNA purification. Cells were collected by centrifugation at 9400 × g for 10 min. at 4 °C and cell pellets were stored at -80 °C until RNA purification.

Growth on mucin was examined by supplementation of the basal medium described above with 0.25 % (v/v) commercial hog gastric mucin (Type III; Sigma-Aldrich), purified by ethanol precipitation as described previously (21).

For fermentation product analysis samples were obtained before inoculation, in mid-exponential phase (~8-10h incubation) and in stationary phase (24h incubation) (Table S1). Carbohydrate degradation and short-chain fatty acid production was determined by high-performance liquid chromatography (HPLC) using a Metacarb 67H column (Varian, Middelburg, The Netherlands).

**Whole-genome transcriptome analysis**

Before total RNA purification, cells were enzymatically lysed using TE-buffer (100mM TRIS and 50mM EDTA, pH 8.0) supplemented with 160,000 U lysozyme and 100 U mutanolysin (both from Sigma-Alderich) during 30 min incubation at 37°C. Genomic DNA was removed by on-column DNase digestion step during RNA purification (DNase I; Roche Diagnostics GmbH, Mannheim, Germany). Yields and RNA-qualities after total RNA purification and success of the rRNA depletion step were assessed using the ExperionTM RNA StdSens Analysis Kit in combination with the ExperionTM System (Bio-Rad Laboratories Inc., Hercules, CA, USA).

rRNA reads were removed with SortMeRNA v1.9 (22) and all included databases. Adapters were trimmed with cutadapt v1.2.1 (23) using default settings except for an increased error value of 20 % for the adapters. Quality trimming was performed with PRINSEQ Lite v0.20.0 (24) with a minimum sequence length of 40 bp and a minimum quality of 30 on both ends of the read and as mean quality. All reads with non-IUPAC characters were discarded as were all reads containing more than three Ns. BAM files were converted with SAMtools v0.1.18 (25) and genome coverage was calculated with BEDTools v2.17.0 (26).

**Growth on Mucin and with Sulfite**

Commercial hog gastric mucin (Type III; Sigma-Alderich) was purified by ethanol precipitation as described previously (Miller et al., 1981). Growth on mucin was examined by supplementation of the basal bicarbonate-buffered medium described in the manuscript with 0.25% (v/v) hog gastric mucin compared to 0.5 % (w/v) D-glucose (Fisher Scientific Inc., Waltham, MA USA). Growth was determined spectrophotometrically by measuring optical density (OD) at 600nm. Growth was checked after overnight incubation (t=16h).

Growth stimulation by sulfite was examined by supplementation of PY medium (Gerritsen et al. 2014) with 30 mM sodium sulfite (Merck KGaA, Darmstadt, Germany) and/or 0.5 % (w/v) D-glucose (Fisher Scientific Inc., Waltham, MA USA). Growth was determined spectrophotometrically by measuring optical density (OD) at 600nm. Growth was checked after overnight incubation (t=16h).

**References**

1. **Koren S, Harhay GP, Smith TP, Bono JL, Harhay DM, McVey SD, Radune D, Bergman NH, Phillippy AM.** 2013. Reducing assembly complexity of microbial genomes with single-molecule sequencing. Genome biology **14:**R101.

2. **Williams D, Trimble WL, Shilts M, Meyer F, Ochman H.** 2013. Rapid quantification of sequence repeats to resolve the size, structure and contents of bacterial genomes. BMC genomics **14:**537.

3. **Boisvert S, Laviolette F, Corbeil J.** 2010. Ray: simultaneous assembly of reads from a mix of high-throughput sequencing technologies. Journal of computational biology : a journal of computational molecular cell biology **17:**1519-1533.

4. **Hernandez D, Francois P, Farinelli L, Osteras M, Schrenzel J.** 2008. De novo bacterial genome sequencing: millions of very short reads assembled on a desktop computer. Genome research **18:**802-809.

5. **Delcher AL, Phillippy A, Carlton J, Salzberg SL.** 2002. Fast algorithms for large-scale genome alignment and comparison. Nucleic Acids Res **30:**2478-2483.

6. **Gao S, Sung WK, Nagarajan N.** 2011. Opera: reconstructing optimal genomic scaffolds with high-throughput paired-end sequences. Journal of computational biology : a journal of computational molecular cell biology **18:**1681-1691.

7. **Langmead B, Trapnell C, Pop M, Salzberg SL.** 2009. Ultrafast and memory-efficient alignment of short DNA sequences to the human genome. Genome biology **10:**R25.

8. **Boetzer M, Henkel CV, Jansen HJ, Butler D, Pirovano W.** 2011. Scaffolding pre-assembled contigs using SSPACE. Bioinformatics **27:**578-579.

9. **Boetzer M, Pirovano W.** 2012. Toward almost closed genomes with GapFiller. Genome biology **13:**R56.

10. **Langmead B, Salzberg SL.** 2012. Fast gapped-read alignment with Bowtie 2. Nature methods **9:**357-359.

11. **Lang JM, Darling AE, Eisen JA.** 2013. Phylogeny of bacterial and archaeal genomes using conserved genes: supertrees and supermatrices. PLoS One **8:**e62510.

12. **Darling AE, Mau B, Perna NT.** 2010. progressiveMauve: multiple genome alignment with gene gain, loss and rearrangement. PloS one **5:**e11147.

13. **Alikhan NF, Petty NK, Ben Zakour NL, Beatson SA.** 2011. BLAST Ring Image Generator (BRIG): simple prokaryote genome comparisons. BMC genomics **12:**402.

14. **Overbeek R, Fonstein M, D'Souza M, Pusch GD, Maltsev N.** 1999. The use of gene clusters to infer functional coupling. Proc Natl Acad Sci U S A **96:**2896-2901.

15. **Tatusov RL, Koonin EV, Lipman DJ.** 1997. A genomic perspective on protein families. Science **278:**631-637.

16. **Nielsen N, Engelbrecht J, Brunak S, von Heijne G.** 1997. Identification of prokaryotic and eukaryotic signal peptides and prediction of their cleavage sites. Protein Engineering **10**.

17. **Latendresse M, Krummenacker M, Trupp M, Karp PD.** 2012. Construction and completion of flux balance models from pathway databases. Bioinformatics **28:**388-396.

18. **Suthers PF, Dasika MS, Kumar VS, Denisov G, Glass JI, Maranas CD.** 2009. A genome-scale metabolic reconstruction of *Mycoplasma genitalium*, iPS189. PLoS computational biology **5:**e1000285.

19. **Heinemann M, Kummel A, Ruinatscha R, Panke S.** 2005. In silico genome-scale reconstruction and validation of the Staphylococcus aureus metabolic network. Biotechnology and bioengineering **92:**850-864.

20. **Fischer JD, Holliday GL, Thornton JM.** 2010. The CoFactor database: organic cofactors in enzyme catalysis. Bioinformatics **26:**2496-2497.

21. **Miller RS, Hoskins LC.** 1981. Mucin degradation in human colon ecosystems. Fecal population densities of mucin-degrading bacteria estimated by a "most probable number" method. Gastroenterology **81:**759-765.

22. **Kopylova E, Noe L, Touzet H.** 2012. SortMeRNA: fast and accurate filtering of ribosomal RNAs in metatranscriptomic data. Bioinformatics **28:**3211-3217.

23. **Martin M.** 2011. Cutadapt removes adapter sequences from high-througput sequencing reads. EMBnet.journal **17:**10-12.

24. **Schmieder R, Edwards R.** 2011. Quality control and preprocessing of metagenomic datasets. Bioinformatics **27:**1783-1785.

25. **Li H, Handsaker B, Wysoker A, Fennell T, Ruan J, Homer N, Marth G, Abecasis G, Durbin R, Genome Project Data Processing S.** 2009. The Sequence Alignment/Map format and SAMtools. Bioinformatics **25:**2078-2079.

26. **Quinlan AR, Hall IM.** 2010. BEDTools: a flexible suite of utilities for comparing genomic features. Bioinformatics **26:**841-842.

27. **Gerritsen J, Fuentes S, Grievink W, van Niftrik L, Tindall BJ, Timmerman HM, Rijkers, GT, Smidt H. 2014.** Characterization of *Romboutsia ilealis* gen. nov., sp. nov., isolated from the gastro-intestinal tract of a rat, and proposal for the reclassification of five closely related members of the genus *Clostridium* into the genera *Romboutsia* gen. nov., *Intestinibacter* gen. nov., *Terrisporobacter* gen. nov. and *Asaccharospora* gen. nov. Int J Syst Evol Microbiol **64:**1600-1616.

28. **Miller RS, Hoskins LC . 1981.** Mucin degradation in human colon ecosystems. Fecal population densities of mucin-degrading bacteria estimated by a “most probable number” method. *Gastroenterolog*y **81** (4): 759-765.
