# Supplementary figures and images for: Genomic and functional analysis of Romboutsia ilealis CRIBT reveals adaptation to the small intestine
Source: PeerJ. 2017 Sep 11;5:e3698. doi: 10.7717/peerj.3698 (PMC5598433; doi:10.7717/peerj.3698)

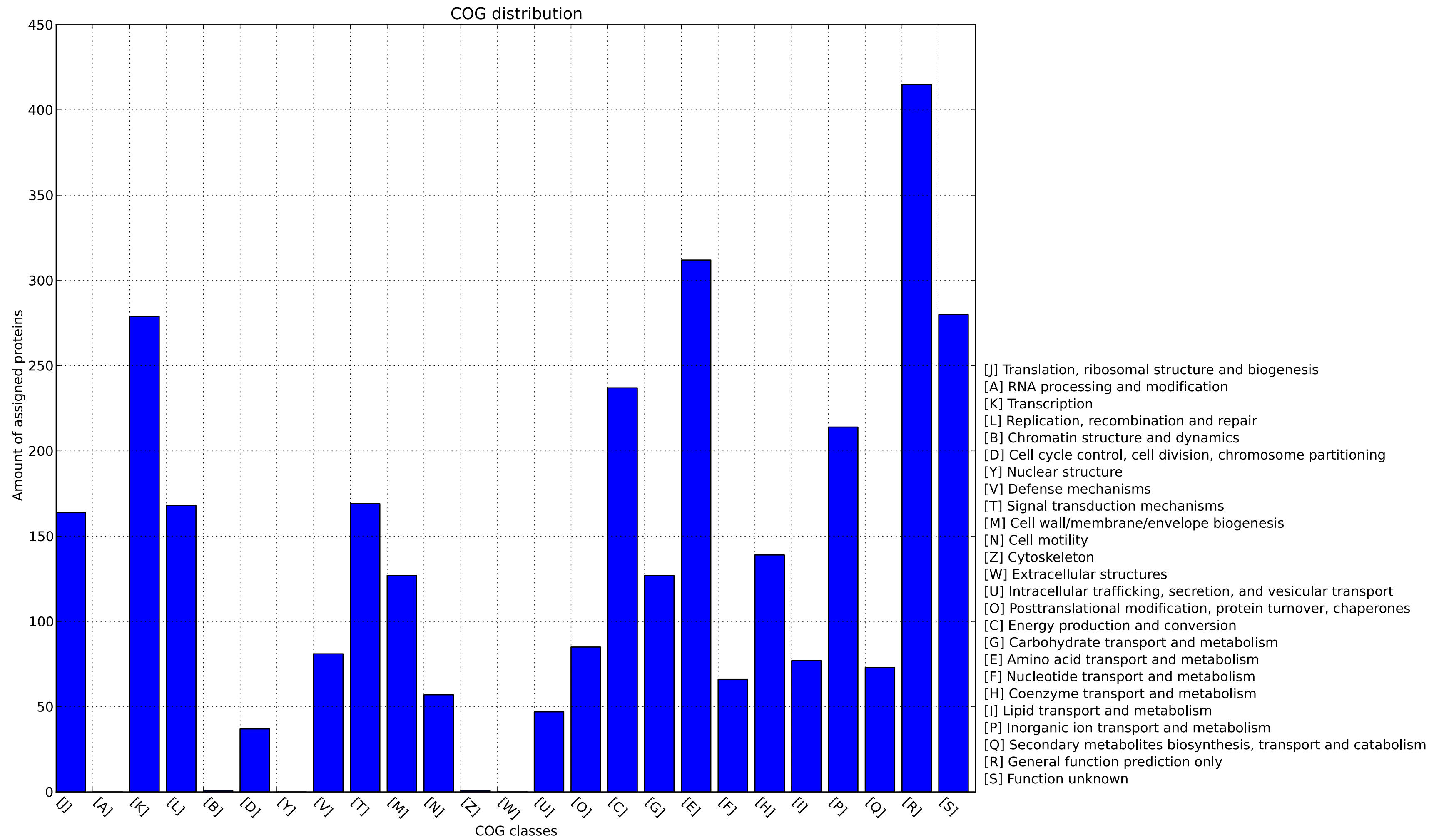

Supplement: Figure S1 [file peerj-05-3698-s002.pdf]

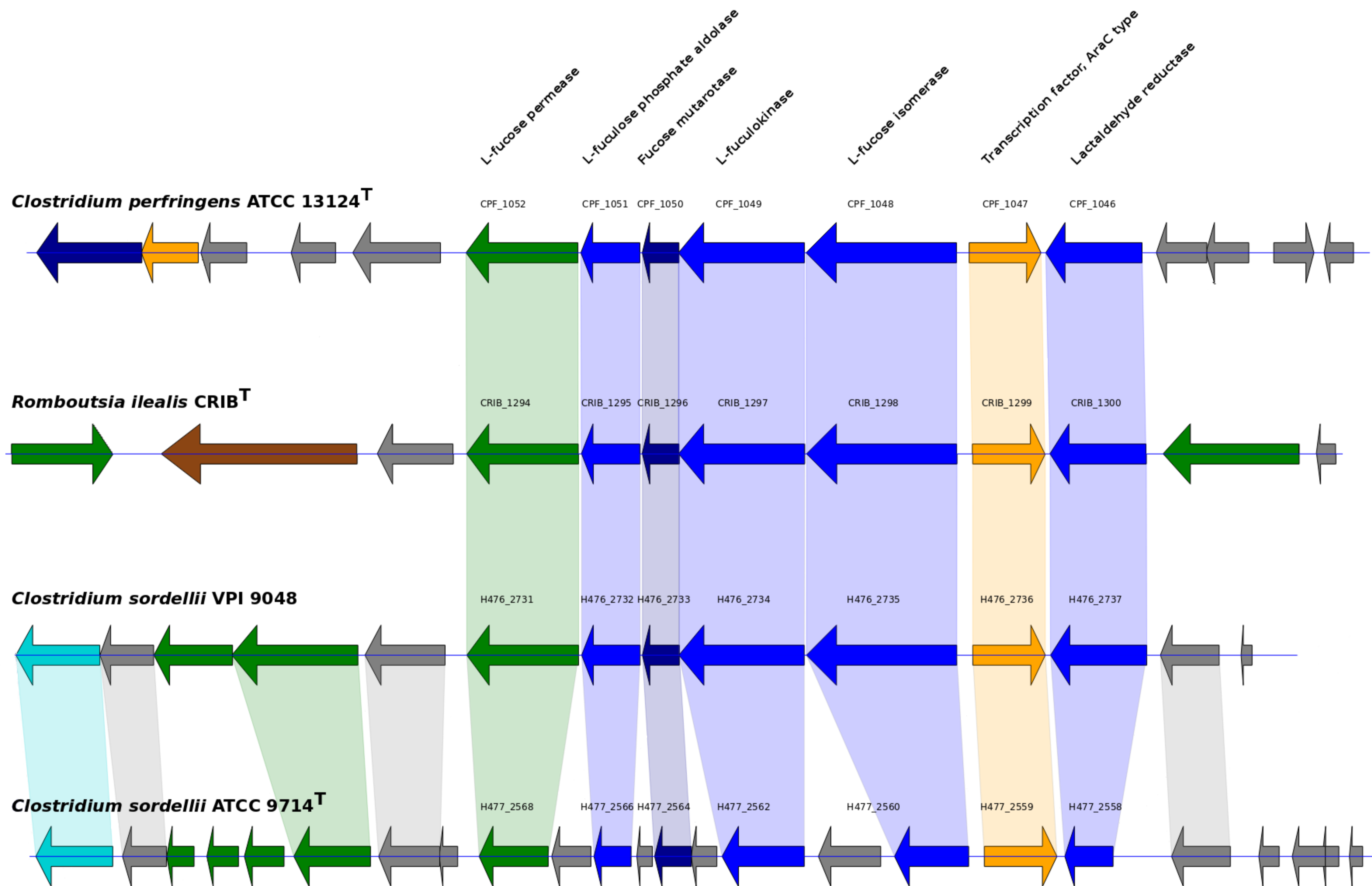

Supplement: Figure S2 — In the current annotation of C. sordellii ATCC 9714T open reading frame prediction seems to be suboptimal, with a considerable amount of potentially wrong stop codons. Genes are color-coded by predicted function: transporter (green), metabolic enzyme with EC number (blue), metabolic enzyme with preliminary EC number/without assigned EC number (dark blue), transcriptional regulator (yellow), hypothetical/unknown protein (grey), protein involved in DNA processing (brown), protein involved in vitamin metabolism (light blue). [file peerj-05-3698-s003.pdf]

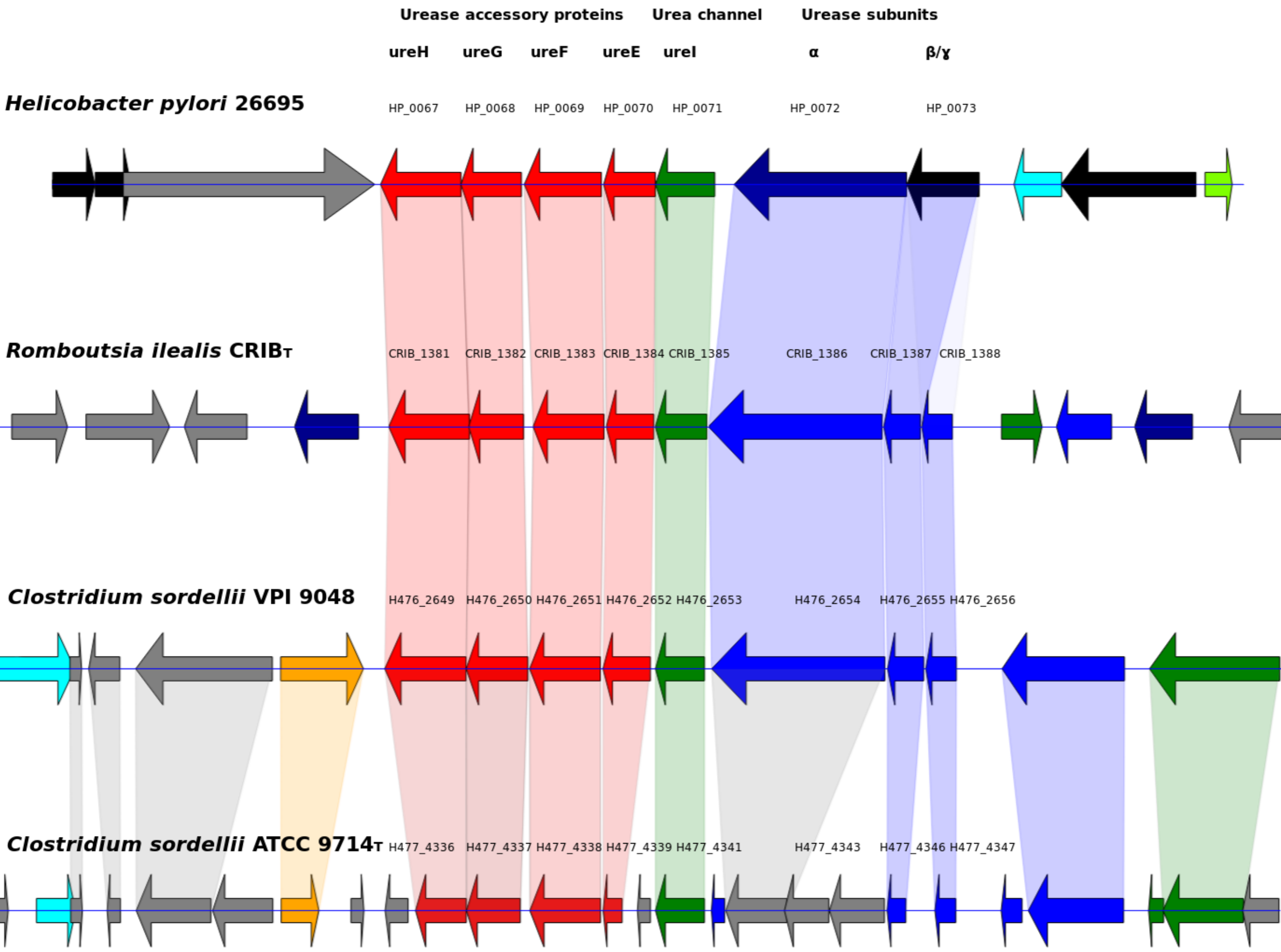

Supplement: Figure S3 — In the current annotation of C. sordellii ATCC 9714T open reading frame prediction seems to be suboptimal, with a considerable amount of potentially wrong stop codons. Genes are color-coded by predicted function: urease accessory proteins (red), transporter (green), metabolic enzyme with EC number (blue), metabolic enzyme with preliminary EC number/without assigned EC number (dark blue), transcriptional regulator (yellow), hypothetical/unknown protein (grey), protein involved in vitamin metabolism (light blue), ribosomal proteins (green). [file peerj-05-3698-s004.pdf]
